# Analyzing the effects of social distancing on the COVID-19 pandemic in Korea using mathematical modeling

**DOI:** 10.4178/epih.e2020064

**Published:** 2020-09-07

**Authors:** Sunhwa Choi, Moran Ki

**Affiliations:** Department of Cancer Control and Population Health, Graduate School of Cancer Science and Policy, National Cancer Center, Goyang, Korea

**Keywords:** Coronavirus, Mathematical model, Outbreak, Reproductive number, Social distancing, Korea

## Abstract

**OBJECTIVES:**

During the 6 months since the first coronavirus disease 2019 (COVID-19) patient was diagnosed in Korea on January 20, 2020, various prevention and control measures have been implemented according to the COVID-19 epidemic pattern. Therefore, this study aimed to estimate the reproductive numbers (R) for each epidemic stage to analyze the effects of the preventive measures and to predict the COVID-19 transmission trends.

**METHODS:**

We estimated the transmission rates for each epidemic stage by fitting a COVID-19 transmission model, based on a deterministic mathematical model, to the data on confirmed cases. The effects of preventive measures such as social distancing by time period were analyzed, and the size and trends of future COVID-19 outbreaks were estimated.

**RESULTS:**

The value of R was 3.53 from February18, 2020 to February 28, 2020, and the mean R reduced to 0.45 from March 14, 2020 to April 29, 2020, but it significantly increased to 2.69 from April 30, 2020 to May13, 2020 and it was maintained at 1.03 from May 14, 2020 to July 23, 2020.

**CONCLUSIONS:**

According to the estimated R, it had fallen to below 1 and was maintained at that level owing to the isolation of infected persons by the public health authorities and social distancing measures followed by the general public. Then, the estimated R increased rapidly as the contact among individuals increased during the long holiday period from April 30, 2020 to May 5, 2020. Thereafter, the value of R dropped, with the continued use of preventive measures but remained higher than 1.00, indicating that the COVID-19 outbreak can be prolonged and develop into a severe outbreak at any time.

## INTRODUCTION

As of July 23, 2020, 15,069,897 confirmed coronavirus disease 2019 (COVID-19) cases and 619,746 COVID-19-related deaths have been reported worldwide; in Korea, 13,938 confirmed cases and 297 deaths have been reported [1,2].

The first confirmed case in Korea was reported on January 20, 2020. From then till February 17, 2020 there were sporadic outbreaks linked to imported cases, resulting in 30 new confirmed cases over an approximately 30-day period. Subsequently, the 31st case was confirmed on February 18, 2020, and from that day to March 7, 2020 large-scale community transmission was observed in Daegu and North Gyeongsang Province, along with sporadic cases being reported in other regions. On February 23, 2020, the quarantine authority implemented active response measures, including upgrading the infectious disease crisis alert level to “serious,” in an effort to reduce the outbreak of COVID-19. The number of cases due to the large-scale community transmission in Daegu and North Gyeongsang Province subsequently reduced. However, from March 8, 2020, the number of imported cases from Europe and America began to increase while sporadic outbreaks continued in other communities. In response, the quarantine authority implemented a high-intensity social distancing policy from March 22, 2020 to April 19, 2020. Between April 20, 2020 and May 5, 2020, a more relaxed social distancing policy was implemented. While the number of imported cases continued to increase, community transmission cases were decreasing. At the time, the number of daily new confirmed cases, excluding imported cases, was in the single digit, or none at all. However, during the long holiday period between April 30, 2020 and May 5, 2020, human-to-human contact increased, and so did the number of new confirmed cases, with many cases linked to Itaewon club cluster. On June 15, 2020 alone, 37 new confirmed cases were reported, and the number of daily new confirmed cases remained between 30 and 50 thereafter.

Accordingly, this study aimed to provide important information in deciding interventions and policies needed, both immediately and in the future, by analyzing the effects of social distancing through the estimation of transmission rates by period and forecasting the future trend in the number of confirmed COVID-19 cases.

## MATERIALS AND METHODS

**Figure 1.**
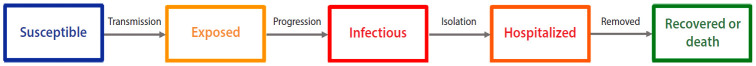
Coronavirus disease 2019 (COVID-19) pandemic based on a deterministic SEIHR model: susceptible group (S), exposed group (E), infectious group (I), hospitalized group (H), and recovered (or dead) group (R).

A previously developed model was used as the COVID-19 pandemic model [3]. The population was divided into five groups: susceptible (S), exposed (E), infectious (I), hospitalized (H), and recovered (R) (Figure 1). The parameters of disease transmission are listed in Table 1.

The model constant *β* refers to the transmission rate. A susceptible individual may be exposed to an infected individual, and this individual may then present with symptoms after the latent period. The constant *κ* refers to the progression rate of COVID-19 symptoms, while 1/*κ* represents the average latent period of the disease. A symptomatic individual is admitted to a hospital for confirmatory diagnosis and isolation and is subsequently moved to the recovered group after going through the recovery period. The constant *α* refers to the isolation rate, while 1/*α* represents the average time between the presentation of symptoms and confirmed diagnosis – this represents the infectious or transmissible period. The constant *γ* refers to the recovery rate among isolated patients, while 1/*γ* represents the average isolation period, until recovery.

To estimate the transmission rate, data regarding daily confirmed cases (from February 18, 2020 to July 23, 2020), by analyzing data published by the Korea Centers for Disease Control and Prevention (KCDC), and the 2019 population data, from the Korean Statistical Information Service, was used for the total population of Korea [2,5]. Starting from April 1, 2020, everyone entering Korea from a foreign country was placed under a 2-week self-quarantine. Accordingly, imported cases between April 1, 2020 and July 23, 2020 were excluded from analysis and only domestic cases were analyzed.

For estimation of transmission rate, the least squares fitting method was used, which minimizes the sum of square error in cumulative confirmed cases (*x*) and modeling result (*αI*). In other words, transmission rate with minimized Σ*i*(*cumulative* [*x_i_*] *γ*[*αI*]*_i_*)^2^ was estimated using the “lsqcurvefit” package of Matlab.

The period between February 18, 2020 and July 23, 2020 was divided into five intervals, considering the implemented policies and change in data pattern, to estimate the transmission rate per interval. The social distancing policy was implemented on February 29, 2020. A 5-day rotation system for purchasing masks was implemented, and the list of countries subject to special entry procedure, starting with Japan, was brought into effect on March 9, 2020. The World Health Organization declared COVID-19 a global pandemic on March 11, 2020. Following this, a strict social distancing policy was implemented between March 22, 2020 and April 19, 2020, which was relaxed from April 20, 2020. However, after the long holiday between April 30, 2020 and May 5, 2020, a transition was made to distancing in daily life, starting from May 6, 2020. Once this transition was made, the number of confirmed cases rose sharply, with most cases reported in the capital region. Accordingly, a recommendation was issued on May 8, 2020 for clubs and bars to suspend their operation and a 2-week prohibition was ordered for mass gathering in bars in Seoul and Gyeonggi region, put into effect from May 9, 2020 and May 10, 2020, respectively. However, because such policies are not effective immediately, the intervals were divided based on dates on which distinct changes in data patterns appeared around the policy implementation dates. With respect to changes in the data pattern, the dates on which the rate of change of slope of cumulative data (second derivative) changed signs or changed significantly were selected. Consequently, transmission rate was estimated using the following five intervals (month/day/year): Interval 1: February 18, 2020 to February 28, 2020; Interval 2: February 29, 2020 to March 13, 2020; Interval 3: March 14, 2020 to April 29, 2020; Interval 4: April 30, 2020 to May 13, 2020; and Interval 5: May 14, 2020 to July 23, 2020.

Based on the estimated transmission rate, the scale of a future COVID-19 pandemic and the outbreak pattern were estimated according to the reproductive numbers (R) value for each scenario. Also, July 24, 2020 was selected as the date for applying the scenario.

### Ethics statement

This research is based on data which is open to public. Neither ethical approval of an institutional review board nor written informed consent were required.

## RESULTS

**Figure 2.**
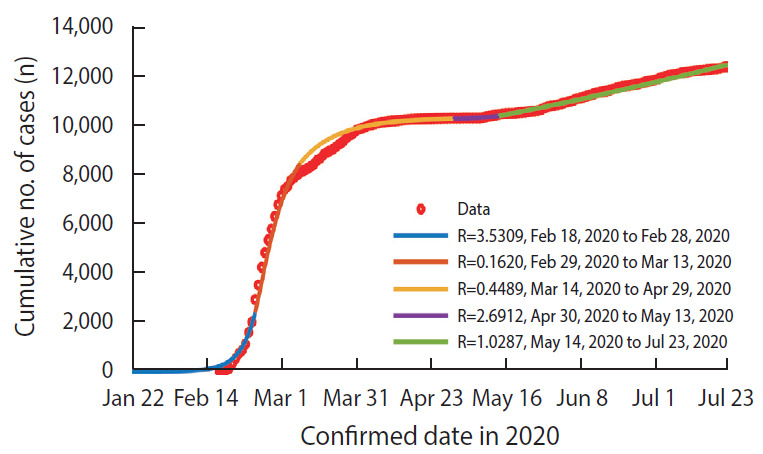
Number of cumulative confirmed coronavirus disease 2019 (COVID-19) cases in Korea according to confirmed date between February 18 and July 23 (red dotted line) and model fitting curve for estimating reproductive numbers (R) (solid line); Interval 1 (February 18 to February 28) represents the Daegu/North Gyeongsang Province outbreak and Interval 4 (April 30 to May 13) represents the outbreak originating from Itaewon.

The results showed that the mean R value in the early stage of COVID-19 community transmission in Korea (based on confirmed date, February 18 to February 28, 2020) was 3.53, which decreased to 0.45 during Interval 3 (March 14 to April 29, 2020). However, there was a large increase in the mean R value to 2.69 during Interval 4 (April 30 to May 13, 2020), while the mean R value was maintained at 1.03 during the final interval. As the duration of the final interval (Interval 5) of the simulation became longer, the mean R value gradually decreased (Appendix 1). The mean R value gradually decreased according to the fitting duration of Interval 5, from R=1.05 (based on confirmed date from May 14 to June 22, 2020) to R=1.03 (based on confirmed date from May 14 to July 23, 2020). On the basis of these results, the scale of a future coronavirus pandemic and the outbreak pattern were estimated (Figure 2).

The scenarios were constructed as follows:

Scenario 1: Assuming that the current R (1.03) value is maintained; Scenario 2: Assuming a 30% increase relative to the current R value due to continued sporadic outbreaks of small-scale cluster infections (R=1.34); Scenario 3: Assuming the R value returns to a similar level (0.45) as before the start of the long holiday (April 30, 2020) owing to continued quarantine and control measures.

If the current trend continues (R value is maintained at 1.03), there should be no significant changes in the pattern of COVID-19 outbreaks since the R value is very close to 1.00. Currently, the number of daily confirmed cases ranges from 20 to 30. Therefore, if the current trend holds, then the number of daily confirmed cases is predicted to be 35 on August 6, 2020 (2-week later) and 37 on August 20, 2020 (4-week later). However, it is possible that the R value will continue to increase due to continued sporadic small-scale cluster infections occurring around the capital region. If the R value increases by 30% relative to the current level and becomes 1.34, then the number of confirmed cases 2 weeks and 4 weeks later would be 53 and 91, respectively. Therefore, despite the R value being smaller than that of Interval 4, the number of daily confirmed cases will be much higher than that of Interval 4.

**Figure 3.**
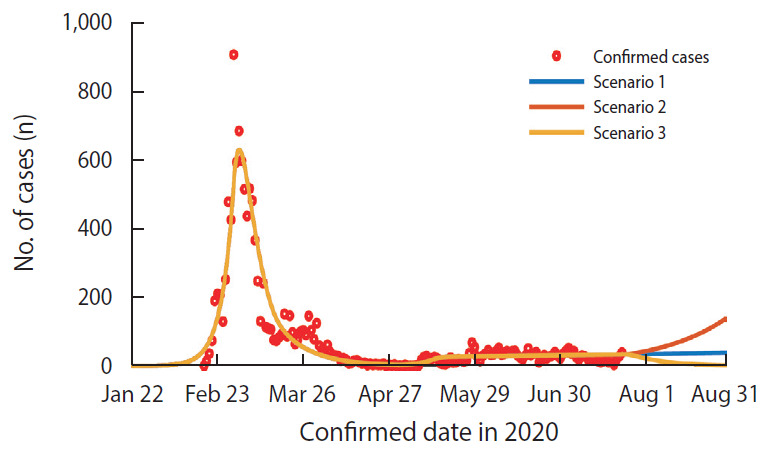
Number of daily confirmed cases by coronavirus disease 2019 (COVID-19) pandemic scenarios. Scenario 1 is when the current R value (=1.03) is maintained; Scenario 2 is when the R value increases by 30% to R=1.34; and Scenario 3 is when the R=0.45 (Interval 3 level) is maintained.

If the R value returns to 0.45, a similar level as that before the start of the long holiday (April 30, 2020) owing to continued quarantine and control measures, then the number of daily confirmed cases will gradually decrease to 15 on August 6, 2020 and 5 on August 20, 2020. Accordingly, it is estimated that the number of daily confirmed cases could reach ≤ 10 by August 12, 2020 and ≤ 1 by September 3, 2020 (Table 2 and Figure 3).

## DISCUSSION

Since the 31st confirmed case of COVID-19 in Korea on February 18, 2020, community outbreaks of COVID-19 have continued to the present time (as of July 23, 2020). Despite major outbreaks in Daegu/North Gyeongsang Province in February and March, the number of cases decreased quickly owing to the aggressive response by the quarantine authority and the active following of social distancing measures by the citizens of Korea. The R value, which was 3.53 at the initial stage of the outbreak, decreased rapidly to 0.16 as the number of confirmed cases in Daegu/North Gyeongsang Province decreased. However, the value increased again due to an increase in the number of imported cases in mid-March and increased to a level slightly greater than the previous level due to sporadic outbreaks in other regions; however, it was still below 1.00, at 0.45. The number of daily confirmed cases also decreased to single-digit numbers at the time, giving the appearing of the pandemic subsiding. However, during the long holiday period between April 30, 2020 and May 5, 2020, human-to-human contact increased and the transition was made from social distancing (level 2 social distancing) to distancing in daily life (level 1 social distancing); following this, the R value increase 2.69, and the number of confirmed cases increased, mostly around the capital region. Owing to intervention policies implemented by the quarantine authority, including implementation of a 2-week mass gathering prohibition order for bars in Seoul (May 9, 2020) and Gyeonggi region (May 10, 2020), the R value decreased again to 1.03 from May 14, 2020 to July 23, 2020, but the value remained> 1.00. If the current R value is maintained, the scale of outbreaks will not increase rapidly, but it may increase gradually, thus making it difficult for there to be an end in sight for the COVID-19 pandemic. Now is an important time to determine whether there will be a recurrence of COVID-19 outbreak or not. As the duration of the final interval (Interval 5) of the simulation became longer, the mean R value estimated up to that point gradually decreased. Therefore, if the outbreak trend remains similar to the current trend, there will be a time when the mean R value falls below 1.00. However, if sporadic clusters of infections continue and increase in scale, then the R value may increase. As shown by the simulation results, if the R value increases to 1.34, the number of daily confirmed cases could be higher than that seen during Interval 4 (April 30, 2020 to May 13, 2020), despite the R value being lower than that of Interval 4 (2.69).

It is estimated that the number of daily confirmed case on August 12, 2020 could be in the single digits if the R value is reduced to 0.45, which was the level when high-intensity social distancing was implemented. For this, it is necessary to have quarantine and control measures in place that can ensure reduced contact with infected patients, including re-implementation of the social distancing policy. With the capital region experiencing continued outbreaks, starting from the Itaewon cluster, Seoul and Gyeonggi Province have already attempted to reduce human-to-human contact by issuing prohibition orders for mass gathering.

The findings in this study were derived using confirmed cases, excluding imported cases based on the assumption that people entering Korea cannot transmit infection since a mandatory 2-week self-quarantine for all incoming travelers was implemented starting April 1, 2020. However, if circumstances in the future change and self-quarantine for foreign travelers becomes difficult due to their large numbers, then the pattern of outbreaks may also change.

In conclusion, the R value, which was as high as 3.53 during the Daegu/North Gyeongsang Province outbreak, reduced to < 1.00 and stayed at this rate through contact tracing and tracking of infected patients by the quarantine authority and adherence to the social distancing policy by the citizens of Korea. However, the outbreaks continued as the R value has increased to > 1.00 due to increased human-to-human contact from relaxed social distancing, and the gradual transition to distancing in daily life, in conjunction with the long holiday period between April 30, 2020 and May 5, 2020. In addition, people have become jaded by the COVID-19 quarantine and control measures, as the pandemic has persisted for over 6 months, and this could lead to increase in negligence in complying with the rules for COVID-19. Therefore, along with the continued efforts made by the quarantine authority, more active public participation in quarantine and control efforts are needed, such as mask wearing, frequent handwashing, and continued social distancing.

## Figures and Tables

**Table 1.** Parameters of coronavirus disease 2019 (COVID-19) transmission model

Symbol	Description	Value	Reference
*β*	Transmission rate^1^	Interval 1 3.531	Data fitted
		Interval 2 0.162	
		Interval 3 0.449	
		Interval 4 2.691	
		Interval 5 1.029	
*κ*	Progression rate (or symptoms progression rate)	1/4	[3,4]
*α*	Isolation rate	1/4	[3,4]
*γ*	Removal rate for isolated individuals (or recovery rate)	1/14	[3,4]

Interval 1, Feb18-Feb 28; Interval 2, Feb 29-Mar 13; Interval 3, Mar 14-Apr 29; Interval 4, Apr 30-May 13; and Iterval 5, May 14-Jul 23 in 2020.

1 Transmission rates are estimated values from the results.

**Table 2.** Changes in the number of daily confirmed case by coronavirus disease 2019 (COVID-19) pandemic scenarios^1^

No. Scenario	R	No. of daily confirmed cases 2 wk later/cumulative confirmed cases (Aug 6, 2020)	No. of daily confirmed cases 4 wk later/cumulative confirmed cases (Aug 20, 2020)	Point of reaching daily confirmed case ≤10	Point of reaching daily confirmed case ≤1
1. Current	1.03	35 (12,973)	37 (13,482)	-	-
2. 30% increase from current level	1.34	53 (13,073)	91 (14,075)	-	-
3. Maintain at Interval 3 level	0.45	15 (12,831)	5 (12,946)	Aug 12, 2020	Sep 3, 2020

R, reproductive numbers.

1 Imported cases were not included in the daily confirmed cases, and cumulative cases estimated by simulation.

## SUPPLEMENTARY MATERIALS

Korean version is available at http://www.e-epih.org/.
