# Supplementary material for: Analyzing the effects of social distancing on the COVID-19 pandemic in Korea using mathematical modeling
Source: Epidemiol Health. 2020 Sep 7;42:e2020064. doi: 10.4178/epih.e2020064 (PMC7871167; doi:10.4178/epih.e2020064)
Supplement: Supplementary file 1 [file epih-42-e2020064-suppl.docx]

**Analyzing the effects of social distancing on the COVID-19 pandemic in Korea using mathematical modeling**(코로나-19 대유행에 미친 사회적 거리두기 효과 분석: 수학적 모델링을 활용하여)

Sunhwa Choi^1^, Moran Ki^1^

^1^Department of Cancer Control and Population Health, Graduate School of Cancer Science and Policy, National Cancer Center, Goyang, Korea

최선화, 기모란

국제암대학원대학교 암관리학과, 국립암센터, 고양, 한국

**Correspondence to:** Moran Ki, moranki@ncc.re.kr

ORCID

Sunhwa Choi, http://orcid.org/[0000-0002-6608-6981](http://orcid.org/0000-0002-6608-6981)

Moran Ki, http://orcid.org/0000-0002-8892-7104

초록:

목적(Objectives): 한국에서 코로나-19 첫 환자가 2020년 1월 20일 진단된 이래 6개월 동안 코로나 유행양상에 따라 사회적 거리두기를 포함한 여러 방역정책이 시행되었다. 이에 수학적 모델링을 활용하여 시기별 감염재생산수(reproductive number, $\mathcal{R}$)를 추정하고 그에 따른 방역정책의 효과를 분석하여 향후 유행의 규모와 유행 종료 시점 등 코로나-19 확산 양상을 예측하고자 한다.

방법(Methods): 결정론적 수학적 모델(SEIHR)에 기반한 코로나-19 확산 모델에 확진자 데이터를 피팅하여 시기별 전파율을 추정하였다. 시기별 사회적 거리두기 등의 방역정책의 효과를 분석하고, 향후 코로나 유행 규모와 추이를 추정하였다.

결과(Results): 코로나-19 지역사회 전파 초반 (확진일 기준 2월18일부터 2월28일까지)의 $\mathcal{R}$ 값은 3.53, 그 이후, 3월14일부터 4월30일까지의 평균 $\mathcal{R}$ 값은 0.45로 감소하였으나, 4월30일부터 5월13일까지의 평균 $\mathcal{R}$ 값은 2.69로 크게 증가하였다. 이후5월14일부터 7월23일 현재까지 R값은 1.03으로 유지되고 있다.

결론(Conclusions): 추정된 감염재생산수에 의하면 방역 당국의 감염자/접촉자 격리 및 시민의 사회적 거리 두기를 통해서 감염재생산수가 1이하로 감소하여 잘 유지되어 오다가 4월30일부터 5월5일까지의 긴 연휴 기간을 통해 사람들 간의 접촉이 증가하면서 감염재생산수가 급격히 증가하였고, 그 후, 지속적인 방역조치로 인해 $\mathcal{R}$**이** 감소하였으나, 여전히 1보다 큰 값으로 나타나, 코로나-19유행이 지속되고 있으며 언제라도 다시 큰 유행으로 커질 수 있음을 알 수 있다.

중심단어: 코로나-19(COVID-19), 수학적 모델, 유행, 감염재생산수, 사회적 거리두기, 대한민국

**서론**

2020년 7월 23일 현재 전 세계적으로 15,069,897명의 코로나-19 확진자와 619,746명의 사망자가 보고되었으며, 한국에서는 13,938명의 확진자와 297명의 사망자가 보고되었다 [1, 2].

한국은 2020년 1월20일 첫 감염자 발생을 시작으로 2월17일까지는 해외 유입 확진자와 이와 연관된 사례들이 산발적으로 발생하여 약 30일간 30명의 감염자가 확진되었다. 이후, 2020년 2월18일 31번째 환자를 시작으로 3월7일까지 대구, 경북지역을 중심 대규모 지역사회 감염과 함께, 기타 지역의 산발적 사례들이 발생하였다. 방역당국은 2월 23일 감염병 위기경보를 심각 단계로 상향하는 등의 적극 대응을 시행하여 코로나-19 환자 발생을 감소시키기 위해 노력하였다. 이후 대구/경북 중심의 대규모 지역사회 감염은 소강상태에 접어 들었으나, 3월8일부터 유럽, 미주 등 해외유입 사례가 증가하기 시작하였고, 다른 지역 사회의 산발적인 집단 유행도 지속되었다. 이에 방역당국에서는 3월22일부터 고강도 사회적 거리두기를 실시하였으며, 이는 한차례 연장되어 4월19일까지 실행되었다. 4월20일부터 5월5일까지는 이전보다는 완화된 사회적 거리두기가 실시되었고, 해외유입 사례 발생은 지속해서 일어나고 있었으나, 지역사회 환자 발생이 감소추세였다. 당시 해외 유입 환자를 제외한 하루 확진자가 한 자릿수 유지되거나, 한 명도 발생하지 않는 수준이었다. 그러나 4월30일부터 5월5일까지 긴 연휴기간에 사람 간 접촉이 증가하였고, 이에 따라 이태원 클럽 집단감염 중심으로 하루 발생 확진자 수도 증가하였다. 6월15일 하루 확진자가 37명 발생하였고, 이후에는 꾸준히 하루 30명에서 50명 사이의 환자가 발생하고 있다.

따라서 시기별 전파율 추정을 통해 사회적 거리두기 효과를 분석하고, 향후 코로나-19 확진자 발생 추이를 추정하여, 현재 및 미래에 필요한 중재정책을 결정하는데 중요한 정보를 제공하고자 하였다.

**방법(Methods)**
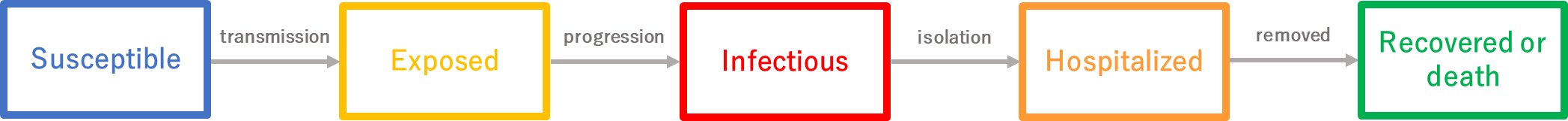


**Fig. 1. 코로나-19 전파 결정론적 SEIHR모델, 감수성자 그룹(S), 잠복 감염자 그룹(E), 감염자 그룹(I), 확진 되어 격리된 그룹(H), 회복자(또는 사망) 그룹(R).**

코로나-19 감염전파 모델은 이전에 개발된 모델을 사용하였다 [3]. 인구 그룹은 질병에 걸릴 수 있는 감수성자 그룹(S), 잠복 감염자 그룹(E), 감염자 그룹(I), 확진 되어 격리된 그룹(H), 회복자 그룹(R) 등 5개의 그룹으로 나누어진다. (**Fig. 1**) **Table 1**은 모델에서 사용된 모수의 정의와 값을 나타낸다.

**Table 1. Parameters of the coronavirus disease 2019 (COVID-19) transmission**

| Symbol | Description | Value | | Reference |
| --- | --- | --- | --- | --- |
| $\beta$ | Transmission rate (감염전파율) | Interval 1 | 3.5309* | data fitted |
|  |  | Interval 2 | 0.1620* |  |
|  |  | Interval 3 | 0.4489* |  |
|  |  | Interval 4 | 2.6912* |  |
|  |  | Interval 5 | 1.0287* |  |
| $\kappa$ | Progression rate (증상발현 진행률) | 1/4 | | [3, 4] |
| $\alpha$ | Isolation rate (격리율) | 1/4 | | [3, 4] |
| $\gamma$ | Removal rate for isolated individuals (회복율) | 1/14 | | [3, 4] |

* 감염전파율은 결과(Results)의 추정된 값

모델 상수 $\beta$는 감염 전파율을 의미하며, 감수성자는 감염자와의 접촉을 통해 감염되어 잠복 감염자가 되고, 잠복기간을 거쳐 잠복 감염자는 증상이 발현된다. 상수 $\kappa$는 코로나-19의 증상발현 진행률을 의미하고 $1/\kappa$은 코로나-19의 평균 잠복기간을 나타낸다. 증상이 발현된 감염자는 의료기관에 내원하여 확진 및 격리가 되고, 회복기간을 거쳐 회복군으로 이동한다. 상수 $\alpha$는 격리율을 의미하며, $1/\alpha$는 증상 발현 이후 확진되기까지의 평균 기간이다. 이는 곧 감염 전파 가능 기간이 된다. 상수 $\gamma$는 격리 감염자의 회복률을 나타내며 $1/\gamma$는 회복되기까지의 평균 격리기간을 의미한다.

감염전파율을 추정하기 위하여 질병관리본부의 보도자료를 분석하여 2020년 2월18일부터 7월 23일까지의 일별 확진자 데이터를 이용하였고, 한국의 총인구 데이터는 국가통계포털의 2019년 인구수를 이용하였다 [2, 5]. 4월1일부터 모든 해외입국자는 2주간 자가격리를 하고 있어서 4월1일부터 7월23일까지 확진자 중 해외입국자는 분석 데이터에서 제외하고 국내 지역사회에서 확진된 자료만 사용하였다.

감염전파율의 추정은 누적 확진자수 데이터($x$)와 모델결과($\alpha I$)의 오차의 제곱의 합이 최소가 되게 하는 최소제곱법(least squares fitting method)을 사용하였다. 즉, $\sum_{i} {(cumulative\left[ x \right]_{i}-\left[ \alpha I \right]_{i})}^{2}$가 최소가 되는 감염전파율을 Matlab의 “lsqcurvefit” 패키지를 사용하여 추정하였다.

2월18일부터 7월23일 구간을 시행된 정책과 데이터의 변화양상을 고려하여 5구간으로 나누어 각 구간별로 감염전파율을 추정하였다. 2월29일 사회적 거리두기 시행, 3월9일 마스크 5부제 시행 및 일본부터 시작된 특별입국절차 대상국이 확대되었으며, 3월12일 WHO에서는 코로나-19 팬데믹 선언을 하였다. 또한, 3월 22일부터 4월 19일까지 강화된 사회적 거리두기를 시행하였고, 4월20일부터는 완화된 사회적 거리두기 시행하였고, 4월30일부터 5월5일까지 연휴기간을 거쳐, 5월6일부터 생활 속 거리두기로 전환하였다. 이 기간을 지나면서 수도권을 중심으로 확진자가 급증함에 따라 5월8일에는 클럽, 유흥주점, 감성주점, 콜라텍 등 운영자제가 권고되었고, 5월9일과 5월10일에는 서울과 경기도에서 각각 유흥주점에 대한 2주간 집합금지 명령이 발동되었다. 하지만, 이러한 정책 시행의 효과가 바로 나타나지는 않기 때문에 정책 시행일 주변으로 데이터 양상의 변화가 두드러지는 날을 기준으로 구간을 나누었다. 데이터 양상 변화는 누적데이터의 기울기의 변화율(두번 미분값)의 부호가 바뀌거나 그 값이 크게 달라지는 날짜를 선택하여 구간1: 2020-02-18~2020-02-28, 구간2: 2020-02-29~2020-03-13, 구간3: 2020-03-14~2020-04-29, 구간4: 2020-04-30~2020-05-13, 구간5: 2020-05-14~2020-07-23으로 5구간으로 나누어 감염전파율을 추정하였다.

추정된 감염전파율을 기반으로 시나리오별 $\mathcal{R}$값에 따라 향후 코로나-19 유행 규모와 발생 양상을 추정하였다. 시나리오 적용일은 7월24일로 설정하였다.

**결과(Results)**

한국에서 코로나-19 지역사회 전파 초반 (확진일 기준 2월18일부터 2월28일까지)의 $\mathcal{R}$ 값은 3.53 이었으나 이후 3월14일부터 4월29일까지의 평균 $\mathcal{R}$ 값은 0.45로 감소되었다. 그러나 4월30일부터 5월13일까지의 평균 $\mathcal{R}$ 값은 2.69로 크게 증가하였고, 5월14일부터 현재까지 $\mathcal{R}$ 값은 1.03으로 유지되고 있다. 시뮬레이션에서 마지막 구간5의 기간이 길어지면서, 그 기간 동안의 추정되는 평균 $\mathcal{R}$ 값이 서서히 감소하고 있는 것으로 나타났다. (Appendix, App_Fig 1) App_Fig 1은 구간 5의 기간에 따른 평균 $\mathcal{R}$ 값을 보여준다. 구간 5에서의 피팅 기간에 따라 $\mathcal{R}$=1.05 (5월14일부터 6월22일 확진자 기준)에서 $\mathcal{R}$=1.03 (5월14일부터 7월23일 확진자 기준)로 서서히 감소하는 경향을 보인다. 이 결과를 바탕으로, 시나리오별 향후 코로나 유행 규모와 발생 변화 양상을 추정하였다. (Fig 2)

**Fig 2.** **한국 2020년2월18일-7월23일까지 확진일에 따른 코로나-19 누적 확진자 수 (붉은색 점)와 감염재생산수(**$\mathcal{R}$**)계산을 위한 모델 피팅 곡선(실선). 구간 1(2/18~2/28)는 대구/경북 유행시기, 구간 4(4/30~5/13)는 이태원발 유행 확산 시기임.**

시나리오 구성은 아래와 같다.

• 시나리오 1: 현재의 $\mathcal{R}$ (1.03)값이 계속 유지된다고 가정

• 시나리오 2: 소규모 집단감염이 산발적으로 지속되어 $\mathcal{R}$이 현재 대비 30% 증가 ($\mathcal{R}=1.34$)

• 시나리오 3: 지속적인 방역조치의 효과로 감염재생산수가 4월30일 연휴 시작 전과 동일한 상황, 즉 R값이 0.45로 돌아가는 것으로 가정

현재의 추세가 계속 유지된다면 ($\mathcal{R}$값이 계속 1.03로 유지), R값이 거의 1에 가까우므로 코로나 확진자 발생 양상은 크게 달라지지 않을 것이다. 현재 코로나 일일 국내 발생 확진자는 20~30명 수준으로, 현재의 추세가 유지되면 2주 후인 8월6일에는 하루 확진자수가 35명, 8월20일에는 하루 확진자수가 37명 발생할 것으로 예측되었다. 그러나, 현재에도 산발적인 소규모 집단감염이 수도권을 중심으로 지속되고 있어, $\mathcal{R}$값이 증가할 가능성이 있다. $\mathcal{R}$값이 현재대비 30% 증가한 1.34 수준이 된다면, 2주 후 하루 확진자와 4주 후 하루 확진자가 각각 53명, 91명으로 구간 4보다 R값은 작지만, 하루 확진자 수는 구간 4 시기보다 더 많이 발생한다.

지속적인 방역조치의 효과로 7월24일부터 $\mathcal{R}$값이 4월30일 연휴 전 상태인 0.45 수준으로 감소한다면 8월6일에는 하루 확진자 15명, 8월20일에는 5명으로 점차 감소할 것이고, 하루 확진자는 8월12일에 10명 이하, 9월3일에 1명 이하가 될 것으로 산출되었다. (Fig 3과 Table 2)

**Fig 3. 코로나19의 유행 시나리오별 일별 확진자 수. 시나리오 1은 현재 R(=1.03)이 지속되는 경우, 시나리오 2는 R=1.34로 현재보다 30% 증가한 경우, 시나리오 3은 구간 3의 R=0.45로 관리되는 경우임.**

**Table 2.** **코로나 19의 유행 시나리오별 확진자 발생수준 변화 양상^1^**

| 시나리오 | $\mathcal{R}$ | 2주 후 하루 확진자수  /누적확진자수  (8월6일) | 4주 후 하루 확진자수  /누적확진자수  (8월20일) | 하루 확진자  10명이하 시점 | 하루 확진자 1명이하 시점 |
| --- | --- | --- | --- | --- | --- |
| 1. 현재 | 1.03 | 35명 (12973명) | 37명 (13482명) | - | - |
| 2. 현재보다  30% 증가 | 1.34 | 53명 (13073명) | 91명 (14075명) | - | - |
| 3. 구간 3 수준으로 관리 | 0.45 | 15명 (12831명) | 5명 (12946명) | 2020-08-12 | 2020-09-03 |

^1^시뮬레이션 결과로 예측된 하루 확진자수와 누적확진자수에 해외유입 사례는 포함되지 않았으며, 국내 발생 사례만 고려하였음.

**토론(Discussion)**

한국에서 코로나-19 31번째 확진자가 2월18일에 진단된 이래 7월23일 현재까지 코로나-19가 지역사회에서 지속해서 유행하고 있다. 2월과 3월에 대구/경북에서 크게 유행하였던 코로나-19가 방역당국의 적극적인 대응과 국민들의 사회적 거리두기 참여로 인해 급격히 감소하였다. 이로 인해, 대구/경북 유행 초기에 3.53이었던 $\mathcal{R}$값은 대구/경북에서 확진자가 급격히 줄어들어 0.16수준까지 떨어졌다가, 3월 중순부터 해외유입으로 인한 사례 발생이 증가하고, 다른 지역 사회의 산발적인 집단 유행이 지속되어 이전의 $\mathcal{R}$값보다 조금 높지만, 여전히 1보다 작은 0.45 수준으로 4월말까지 유지되고 있었다. 당시 일일 확진자 수도 한 자릿수로 감소하면서 유행이 잦아 들고 있었다. 그러나, 4월30일부터 5월5일까지의 긴 연휴 기간을 통해 사람들 간의 접촉이 증가하였고, 또한 사회적 거리두기(사회적 거리두기 2단계)에서 생활 속 거리두기(사회적 거리두기 1단계)로 전환되면서 감염재생산수가 1보다 큰 2.69수준으로 크게 증가한 후, 수도권 중심으로 확진자가 지속적으로 발생하였다. 5월9일 서울과 5월10일 경기도에서 유흥주점 2주간 집합금지 명령이 발동되는 등 방역당국의 중재정책으로 5월14일부터 7월23일 현재까지 $\mathcal{R}$ 값은 1.03으로 감소하였으나, 여전히 1보다 큰 값이 유지되고 있다. 현재의 $\mathcal{R}$수치와 같은 값으로 지속되면 확산규모가 급격히 증가되지는 않지만, 서서히 증가할 가능성이 있고, 코로나-19가 종식되기 어려울 수 있다는 것을 보여준다. 현재가 코로나-19 유행의 재확산 여부를 결정지을 수 있는 중요한 시점이다. 시뮬레이션에서 마지막 구간5의 구간이 길어지면서, 그 기간 동안의 추정되는 평균$\mathcal{R}$값이 서서히 감소하고 있어 코로나 발생 추이가 지금과 유사하게 나타난다면 평균 $\mathcal{R}$값은 1이하로 내려가는 시점이 나타날 것이다. 그러나 집단감염이 산발적으로 지속되고, 집단감염의 규모가 커지면 $\mathcal{R}$값이 증가할 가능성이 있다. 시뮬레이션 결과에서 보듯이, $\mathcal{R}$값이 증가하여 1.34 수준이 된다면 4월30일에서 5월13일까지 (구간4)의 $\mathcal{R}$**=2.69** 보다 작음에도 불구하고, 하루 확진자 수는 그 시기보다 더 많이 발생할 수 있다.

지속적인 방역조치의 효과로 고강도 사회적 거리두기를 시행했을 때의 수준인 0.45로 $\mathcal{R}$값이 감소되어야, 8월 12일 하루 확진자가 한 자릿수가 될 것으로 추정되었다. 이를 위하여 사회적 거리두기를 다시 시행하는 것을 포함하여 감염자와 접촉을 감소시킬 수 있는 방역 조치가 요구된다. 이미 서울시와 경기도에서는 이태원에서 시작된 산발적인 집단 유행이 수도권에서 지속되자, 집합금지 행정명령을 시행하는 등 사람들 간의 접촉을 줄이려고 노력하고 있다.

이 연구에서는 4월1일부터 모든 해외입국자를 2주간 자가격리 시행하였기 때문에, 해외입국자는 감염전파를 시킬 수 없다고 가정하고, 해외입국자 데이터는 제외한 확진자 데이터를 사용하여 결과를 도출하였다. 그러나 향후 상황이 변하여 해외입국자가 증가하여 해외입국자 자가격리 관리가 어려워지는 상황이 된다면 유행양상이 변화될 수 있다.

결론적으로, 대구/경북 유행 당시 3.53으로 높았던 감염재생산수는 방역 당국의 감염자/접촉자 추적과 격리 및 시민의 사회적 거리 두기를 통해서 감염재생산수가 1이하로 감소하여 잘 유지되어 오다가 4월30일부터 5월5일까지의 긴 연휴 기간과 완화된 사회적 거리두기, 생활 속 거리두기 등을 통해 사람들 간의 접촉이 증가하면서 감염재생산수가 1보다 커져 현재까지 유행이 지속되고 있다. 생활 속 거리두기(사회적 거리두기 1단계)로의 전환 후, 사람들 간의 접촉은 지속적으로 증가하고 있다. 이와 더불어 코로나 유행이 6개월 넘게 지속되면서 코로나 방역조치에 대한 사람들의 피로도가 높아짐에 따라 코로나-19 생활수칙 준수가 해이해질 수 있다. 그렇기 때문에, 지속적인 방역당국의 노력과 함께 보다 적극적인 국민 참여 방역 운동, 즉 마스크 쓰기, 손씻기, 지속적인 사회적 거리두기 노력이 필요하다.

**Acknowledgement**

코로나-19 유행으로 방역과 치료에 애쓰고 있는 질병관리본부와 보건소 직원들, 현장에서 역학조사를 수행하고 있는 모든 역학조사관들, 전국의 의료진들께 깊이 감사드립니다. 코로나-19 유행을 종식시키기 위하여 개인의 불편을 감수하고 사회적 거리두기에 적극 참여해주시는 국민들께도 감사드립니다. 이 연구과제의 모델링 방법과 결과 해석에 대한 창의적인 제언을 아끼지 않고 해 주신 감염병 모델링 연구팀원들께도 감사드립니다.

This research was supported by Government-wide R&D Fund project for infectious disease research (GFID), Republic of Korea (grant No. HG18C0088).

This research was supported by the Korea National Research Foundation (NRF) grant funded by the Korean government (MEST) (NRF-2019R1A2B5B01101143).

**CONFLICT OF INTEREST**

The authors have no conflicts of interest to declare for this study.

**References**

1. WORLD HEALTH ORGANIZATION, et al. Coronavirus disease 2019 (COVID-19): situation reports. 2020..

2. Korea Centers for Disease Control and Prevention. Current status of COVID-19 outbreak in Republic of Korea. Available from:

<http://ncov.mohw.go.kr/tcmBoardList.do?brdId=&brdGubun=&dataGubun=&ncvContSeq=&contSeq=&board_id=>.

3. CHOI, Sunhwa; KI, Moran. Estimating the reproductive number and the outbreak size of COVID-19 in Korea. Epidemiology and Health, 2020, 42.

4. KI, Moran, et al. Epidemiologic characteristics of early cases with 2019-nCoV disease in Republic of Korea. Epidemiology and Health, 2020, e2020007.

5. Korean Statistical Infromation Service. Population Cencus. Available from:

<http://kosis.kr/statisticsList/statisticsListIndex.do?menuId=M_01_01&vwcd=MT_ZTITLE&parmTabId=M_01_01>

**Appendix**

**App_Fig 1*.* 코로나19 유행 구간5(5월 14일부터 7월 23일)의 마지막 피팅 날짜에 따른 감염재생산수 변화. 예를 들어 5월 14일부터 7월 5일까지 평균 R=1.045**
